# Exogenous Melatonin Improves the Reproductive Outcomes of Yearling Iberian Red Deer (*Cervus elaphus hispanicus*) Hinds

**DOI:** 10.3390/ani11010224

**Published:** 2021-01-18

**Authors:** José Antonio Ortiz, Olga García-Álvarez, Mariano Amo-Salas, Alejandro Maroto-Morales, María Iniesta-Cuerda, María del Rocío Fernández-Santos, Ana Josefa Soler, José Julián Garde

**Affiliations:** 1 Medianilla S.L. Finca Las Lomas, Vejer de la Frontera, Las Lomas, 11179 Cádiz, Spain; jaortiz@gruponetco.com; 2 SaBio IREC (CSIC-UCLM-JCCM), ETSIAM, 02071 Albacete, Spain; jandromaroto@hotmail.com (A.M.-M.); m.iniestacuerda@gmail.com (M.I.-C.); mrocio.fernandez@uclm.es (M.d.R.F.-S.); anajosefa.soler@uclm.es (A.J.S.); julian.garde@uclm.es (J.J.G.); 3 Department of Mathematics, University of Castilla-La Mancha, 13071 Ciudad Real, Spain; mariano.amo@uclm.es

**Keywords:** melatonin implants, *Cervus elaphus hispanicus*, yearling hind, fertility, calves’ weight

## Abstract

**Simple Summary:**

Increasing the reproductive performance of hinds is considered to be a key factor of overall farm deer productivity. In the case of yearling hinds, this aspect becomes more important, as a delay in the pubertal onset will compromise the reproductive performance of the entire herd (decreased fertility), and these yearling hinds will carry this ‘late’ condition throughout their reproductive life. The aim of this study was to explore the use of melatonin implants on yearling Iberian red deer (*Cervus elaphus hispanicus*) hinds to improve their fertility outcomes, advance the calving date and the calves’ weight, and to prevent the negative impact of yearling hinds’ low liveweight on their reproductive outcomes. Melatonin implants (18 mg), administered three-fold (two implants each time) every 30 days before the breeding season, rendered significantly higher fertility rates (regardless of the yearling hind’s weight) and heavier calves, and advanced the calving date in the yearling hinds by 15 days compared to non-treated hinds. In addition, halving the number of yearling hinds that received melatonin provided a similar benefit to a large-scale treatment of the whole herd, which indicates female-to-female stimulation of the ovarian activity. Taken together, this protocol for melatonin treatment simplifies its administration, reduces its costs, and assures the enhancement of the reproductive productivity of the entire farm.

**Abstract:**

The aim of this study was to assess the effect of melatonin implants on the reproductive performance of yearling Iberian red deer (*Cervus elaphus hispanicus*) hinds. It also explored exogenous melatonin administration as a tool to minimize the negative effect of a low yearling hind’s liveweight on their reproductive efficiency. In addition, the effect of melatonin-treated yearling hinds on non-treated hinds was studied in order to provide a practical and economical protocol to improve farms’ productivity. A total of 4520 Iberian red deer hinds belonging to the same farm were included in this study. Melatonin (108 mg/hind) implants were administered three-fold every 30 days before the breeding season. Fertility rates, calves’ weights and calving dates were registered for each hind. The results showed that exogenous melatonin increased significantly (*p* < 0.05) the calves’ weight (32.39 ± 1.07 kg vs. 27.65 ± 1.11 kg for Weight 1_calf_ (July) and 46.59 ± 1.50 kg vs. 41.79 ± 1.54 kg for Weight 2_calf_ (August, at weaning)) and advanced the calving date by 15 days in yearling hinds compared to the non-treated group. In addition, the administration of melatonin implants before the breeding season was able to minimize the negative effect of low yearling hinds’ liveweight (Weight 1_hind_) on their future reproductive outcomes, as the fertility rates increased by 46% and the calves’ weight increased by 7 kg after the melatonin treatment, regardless of the yearlings’ weight. Finally, when both experimental groups (melatonin and non-treated) were kept separate, higher fertility rates (76.73 ± 7.18% vs. 66.94 ± 7.41%) were observed for the melatonin-treated hinds compared to the non-treated hinds. However, when both groups of yearling hinds were maintained together, no significant differences were observed in their fertility outcomes (78.13 ± 21.26% vs. 78.12 ± 23.32%). Therefore, melatonin implants may be used in yearling Iberian red deer hinds as a management tool to improve their reproductive productivity.

## 1. Introduction

Over the past decades, there has been a rapid buildup of farmed deer herds around the world, most of them located in Australia, New Zealand, China and Canada [1,2]. The worldwide demand for deer meat is still growing, and accordingly, Europe is experiencing an increased trend towards deer farming using red deer subspecies, such as Iberian red deer (*Cervus elaphus hispanicus*) [3], which genetically differ from those most commonly used in deer farms.

The Iberian hind is a short day, markedly seasonal breeder, with conception occurring in autumn, and calving occurring in spring and early summer [4,5]. Female red deer reach puberty during the second autumn of life (around 16 months of age) [6], and are constrained by photoperiod and body mass [7,8]. For yearling hinds, the onset of puberty is the major determinant of their lifetime reproductive efficiency [9]. Thus, a failure to attain puberty at 16 months of age seems to account for the low productive outcomes of young red deer hinds, decreasing the fertility of the entire farm [7,10]. This event is undesirable, and has long-term carry-over effects on hinds as well as on calves, due to late calving occur when feeding conditions and the presence of ectoparasites are more unfavorable (summer) [6]. In addition, the weight gain in those ‘late’ calves will not be adequate at winter, negatively impacting the entry into puberty of the born hinds within the next year. Similarly, late born deer will not develop their antlers normally, causing a noticeable delay of their reproductive activity. The aforementioned facts seriously limit the progress of deer farming, since increasing the reproductive performance of the hinds is considered as a key measure of overall farm productivity [11]. However, when a wild species such as Iberian red deer is converted into a livestock species, it is possible to apply reproductive biotechnologies as a management tool to increase their productive outcomes [12].

Melatonin, a hormone mainly secreted by the pineal gland, mediates the effect of the photoperiod on the regulation of the physiological cycle of seasonal breeding animals [13]. The ability of melatonin to stimulate the reproductive activity of females has been previously studied in deer [14,15,16,17,18] and other ruminant species such as goats [19] and sheep [20]. These beneficial effects in small ruminant females have been attributed to its ability to mediate the activation of the hypothalamus–pituitary axis, inducing an increase in GnRH (gonadotropin-releasing hormone), LH (luteinizing hormone) and FSH (follicle-stimulating hormone) secretion, and a reduction of prolactin levels [21,22,23], which enhance pubertal ovulations. Even though melatonin improves reproductive outcomes in deer, there is no information about its application on yearling Iberian hinds, nor on whether melatonin may reduce the influence of body-mass on constraining the entry into puberty. Such a matter is of particular interest to rescue those yearling hinds of low body mass after birth that would otherwise fail to enter puberty in their second year. In addition, it is still unresolved whether selective melatonin administrations might promote the onset of puberty of untreated yearling Iberian hinds, since—in other deer subspecies and other small ruminants—the female-to-female stimulation of ovarian activity has been reported (deer [24], sheep [25], goats [26]). If true, the consequence, from a practical point of view, is huge, as the ovarian activity of yearling hinds could be stimulated with small-scale melatonin treatments.

Since the potential breeding of yearling Iberian red deer hinds has received no scrutiny so far, we hypothesized that, in this red deer subespecies (*Cervus elaphus hispanicus*), the use of melatonin implants in yearling hinds might be a useful reproductive management tool to: (1) advance the calving date (a proxy for puberty attainment), (2) increase the calves’ weight, (3) improve the fertility outcomes, and (4) decrease the yearling hind’s weight threshold to puberty attainment. In addition, a practical use of melatonin implants in deer farming is demonstrated in our study, providing a specific protocol for the administration of melatonin implants in yearling hinds.

## 2. Materials and Methods

### 2.1. Animals

A total of 4520 Iberian red deer hinds were included in this study (yearlings (14–16 months old; *n* = 2532) and adults (>3 years old; *n* = 1982)) between June 1997 and August 2018. The animals were housed in a semi-free regime at Medianilla Red Deer Genetics (Medianilla S.L., Cádiz, Spain) (latitude 36°20′ N, 5°48′ O). Similar health management practices (nutrition, vaccinations, etc.) were applied to all of the animals included in this study (hinds and stags). No ethical approval was deemed necessary; the data provided in our study were obtained after observations of routine procedures performed in the farm, in compliance with the Ethical Principles in Animal Research. Protocols, amendments, and other resources were established and applied according to the guidelines approved by each autonomous government following the RD53/2013 of the Spanish Ministry of Presidency.

### 2.2. Experimental Design

Prior to carrying out the different experiences included in the present study, fertility outcomes after natural mating were explored and compared between adult (*n* = 1982) and yearling (*n* = 588) Iberian red deer hinds for 4 years.

Once the reproductive performance of the adults and yearling hinds was identified, a fixed protocol of melatonin implants (see below for details) was applied to a group of yearling hinds (melatonin) (*n* = 907) for 11 years. Fertility rates and calves’ weights were recorded for each hind. Similar reproductive parameters were simultaneously assessed in a group of non-treated (control) yearling hinds (*n* = 443) (Table 1).

Since a low body weight at birth (proxy to birth date) may constrain the attainment of puberty in hinds, the ability of melatonin implants to minimize the negative impact of yearling hinds’ low weight on reproductive performance was also explored. The weight of the yearling hinds (*n* = 543) was recorded at a fixed date after their birth (in July (Weight 1_hind_), see Section 2.6), quantifying this effect on reproductive productivity (fertility rates, calves’ weight).

Once it was confirmed that melatonin implants improved the reproductive outcomes of yearling Iberian red deer hinds, the effect of the presence of melatonin-treated hinds on the fertility rates of the non-treated hinds (*n* = 57) was evaluated.

### 2.3. Melatonin Treatment

Two implants (MELOVINE^®^/REGULIN^®^; CEVA Animal Health Ltd., Chesham, UK) containing 18 mg melatonin each were administered three-fold subcutaneously at the base of the right ear every 30 days (early June, early July and early August, with the total melatonin dose being 108 mg/hind) by the use of a MELOVINE^®^/REGULIN^®^ implanter gun (CEVA Animal Health Ltd., Chesham, UK). The hinds belonging to the control group received no melatonin treatment. Both groups of yearling hinds (control and Melatonin) remained separated, avoiding any contact between them that may influence the reproductive behavior of the untreated herdmates. However, when female-to-female stimulation was evaluated, both groups were mixed, favoring close contact between the animals. The protocol for the implant administration was selected after preliminary experiments combining a different number of implants (4 vs. 6) and dates of the first administration (May vs. June) (data not shown).

### 2.4. Fertility Outcomes

The stags had no contact with the yearling hinds until the mating period. The hinds were joined with multiple sire stags, at a stag:hind ratio of 1:10, from mid-August to mid-October in each of the study years. The fertility rate for each group (adult, control and melatonin) was calculated as follows: the number of hinds calved/total number of hinds mated. No melatonin implants were administered to the stags.

### 2.5. Parentage Verification

The parentage of the calves was confirmed by genetic tests based on the analysis of highly polymorphic DNA markers, such as microsatellites, knows as STRs (Short Tandem Repeats) that are easily identifiable by PCR (Polymerase Chain Reaction). For this, hair samples (approximately 50–100 hairs) with their follicles were taken from the calves, and were placed into paper envelopes with the identification of the animal. The hair samples were taken from the tail in a dry, clean, and contamination-free area. The collection was carried out whilst changing the gloves at each extraction.

### 2.6. Calves and Hinds’ Weights

The calves’ live bodyweights were recorded at two fixed dates: Weight 1_calf_ (first week of July) and Weight 2_calf_ (at weaning, third week of August) (41 days of length between both weights). Both weights were used to estimate the calving date, as the wild nature of this species and the semi-intensive conditions of the deer farm did not allow us to register the calving date immediately. Thus, in the group of calves born from the control yearling hinds, we calculated the daily weight gain of the calves ((Weight 2_calf_ − Weight 1_calf_) / 41 days). Considering that all the calves were weighed on the same day, we infer that the heavier ones would have been born earlier, and vice versa. Therefore, in order to ascertain the difference in days between the calving date of the melatonin-treated and non-treated yearling hinds, we calculated the difference of Weight 1_calf_ between both groups and divides it by the average daily gain of the untreated hinds’calves (for the average daily gain, see above).

The hinds’ live bodyweights were recorded in a similar way to that which was previously described for the calves, but in the year in which the hind was born. Thus, for the hinds (mothers), Weight 1_hind_ was used as a proxy to the birth date of the hind.

### 2.7. Statistical Analyses

The data were analyzed using IBM SPSS Statistics 24 (IBM Corp., Armonk, NY, USA). The data are shown as mean ± standard error of the mean (SEM). The significance level was set at *p* < 0.05. A linear mixed-effects model in which the group of hinds (adult vs. yearling) or the melatonin treatment (control vs. melatonin) and the stags were considered fixed factors, and the year was considered as a random effect, was performed in order to assess the differences in the fertility rates and calves’ weights. When they were significant, the means were compared using a Bonferroni post hoc test.

In order to study the effect and the relationship of the yearling hind’s weight and the treatment of melatonin on the reproductive performance, linear regression techniques and logistic regressions were applied, depending on whether the dependent variable was quantitative (fertility) or qualitative (calves’ weights). With the logistic regression, in addition, the odds ratio and the adjusted odds ratio were considered to analyze the joint relationship of the variables studied.

## 3. Results

There was no adverse effect (inflammation, infection) at the site of the melatonin implants’ administration. When the fertility rates were explored in adults and yearling Iberian red deer hinds, they were significantly higher (*p* < 0.05) for the adults compared to yearlings (80.22 ± 4.64% vs. 50.68 ± 4.64%).

**Figure 1:**
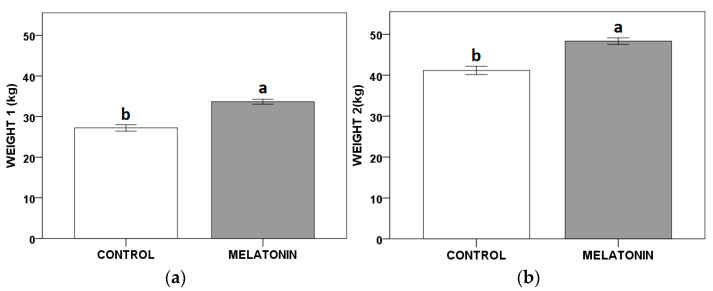
Effect of melatonin implants on the calves’ weights registered at two fixed dates (Weight 1_calf_ (**a**) July; Weight 2_calf_ (**b**) at weaning, August) in yearling Iberian red deer hinds (*Cervus elaphus hispanicus*). Melatonin: the yearling hinds received melatonin implants, administered three-fold (two implants each time) every 30 days before the breeding season; Control: the yearling hinds received no melatonin treatment. Different letters show significant differences (*p* < 0.05) between the experimental groups.

Throughout the studied period, the melatonin treatment resulted in a significant advance of 15 days in the calving date, and an increase of the calves’ Weight 1_calf_ and 2_calf_ (Figure 1) compared to the control yearling hinds.

We observed that, independently, both the weight of the yearling hinds (date of birth) and the treatment with melatonin implants had a positive and significant effect (*p* < 0.05) on the fertility rates (Table 2). In order to quantify this effect, we used a logistic regression to calculate the odds ratio (OR). Thus, we observed that, for each kg that the yearling hind’s weight increased (earliest birth), the fertility increased 1.03 times more (3%), regardless of whether it was treated or not with melatonin implants.

On the other hand, those yearling hinds which received melatonin implants had their fertility increased 1.54 times, regardless of their weight. However, when both factors (weight and melatonin treatment) were considered (AOR, odds ratio adjusted for other factors) at the same time, the yearling’s weight ceased to have a significant effect on fertility, leading to the observation that those females that received melatonin implants increased their fertility 1.46 times regardless of their weight, compared to the yearling hinds belonging to the control group (Table 2).

In addition, when we assessed the effect of the yearling hind’s weight (Weight 1_hind_) and melatonin treatment on the calves’ weights, we noted that, similarly to fertility’s behavior, both factors independently exert a positive effect (*p* < 0.05) on the registered weights. However, when they are considered together, the yearling’s weight again no longer has a significant effect on the calves’ weight. In order to quantify this effect, we used a linear regression, and obtained the following models:

Weight 1_calf_ = 24.51 + (0.095 × yearling hind’s weight) + (7.358 × Treatment *) (1)

Weight 2_calf_ = 40.67 + (0.098 × yearling hind’s weight) + (7.371 × Treatment *) (2)

where * Treatment, 0 = Control and 1 = melatonin implants.

Thus, we can observe that, regardless of the yearling hind’s weight (date of birth) when they received melatonin implants, their calves weighed an average of 7.35 kg (Weight 1_calf_) more than those born from the control females. Accordingly, when the calves’ weights were recorded at weaning (Weight 2_calf_), those from the melatonin treated yearling hinds were 7.37 kg heavier than the calves from the non-treated group. The weight increased in both cases (Weight 1_calf_ and 2_calf_), and it was adjusted for the weight of the yearling hind (Weight 1_hind_).

Finally, when both experimental groups (melatonin and control) were kept separate, higher (*p* < 0.05) fertility rates (76.73 ± 7.18% vs. 66.94 ± 7.41%) were observed for the melatonin-treated hinds compared to the control hinds. However, when both groups of yearling hinds were maintained together, no significant differences were observed in their fertility outcomes (78.13 ± 21.26% vs. 78.12 ± 23.32% for the melatonin- and non-treated, respectively).

## 4. Discussion

In the present study we provide, for the first time, results about the effect of melatonin implants in yearling Iberian red deer (*Cervus elaphus hispanicus*) hinds on their reproductive productivity from a large-scale study carried out in the same farm over an eleven-year period. With our approach, robust conclusions were obtained, as the environmental conditions (latitude) and nutrition regime were the same, the melatonin-treated and non-treated hinds were kept separate (except for female-to-female effect evaluation), and the stags received no melatonin treatment.

It is known that the reproductive performance of yearling red deer hinds is considerably lower than that of adult females [16]. Accordingly, in our conditions, the adult Iberian red deer hinds showed significantly higher fertility compared to the yearling hinds when mating occurred between mid-August and mid-October. A failure in puberty attainment in most of the yearling hinds could account for the differences observed between the two groups of hinds. Extending the mating period could have improved the reproductive outcomes of the yearling Iberian red deer hinds; however, the consequent late calving would in turn carry reductions in the reproductive performance of both the hinds and the calves. The low reproductive productivity of pubertal hinds has been previously reported in other red deer subspecies [17], even in hinds showing good body mass. Thus, the effect of the photoperiod, mediated by melatonin, has been highlighted as a potential factor for the better reproductive efficiency of young hinds.

Different studies have explored the administration of exogenous melatonin to pubertal red deer hinds in order to advance the first estrous, ovulation conception, and parturition; however, the results are highly variable [14,15,16,17,18,27]. Increasing the fertility and advancing the calving date of yearling hinds have advantages for deer breeding, favoring the earlier and higher venison production of calves. Thus, there is a need for ongoing research on exogenous melatonin treatment in order to clarify its effect on the reproductive efficiency of yearling Iberian red deer hinds.

In the present study, the calves’ weights were used to estimate the calving date, inferring that heavier calves were born earlier, and as a proxy for puberty onset. This methodology contrasts with most of the studies carried out up to date [14,15,16,17,18]; our experimental condition—as a semi-extensive production system—hampered the effective recording of the exact time point of such events.

The advancement in the calving season after treatment with melatonin is quite variable between studies (9–68 days) [17,18], mainly due to the differences in the initiation of the administration of the implants as well as the dose of melatonin per hind [28]. Regarding the latter, it has been previously reported in red deer that two melatonin implants (18 mg/implant) elevate the melatonin in the plasma up to the night levels reached within the reproductive season (>100 pg/mL), effectively maintaining them stably for periods of at least 30 days [18]. Accordingly, in our study, when melatonin implants were administered three-fold (two implants each time) every 30 days before the breeding season, the calving date advanced by 15 days compared to the non-treated control. Exogenous melatonin has been successfully used to advance calving in different deer subspecies [18,28]; however, in these studies, the reproductive activity of the stags was enhanced through similar melatonin treatments what could account for an additional stimulus of the females. Notwithstanding this, our work showed how administering melatonin only to yearling hinds allowed earlier calving to occur.

The date of birth of the hind will determine its future reproductive performance. In our latitude, if its entry into puberty delays the moment of mating, parturition will occur when food availability is minimal (summer), putting at risk the survival of the calves and the supply of the high energy demands of the already short lactation period [29,30]. Our results showed that yearling hinds’ weights (as a proxy to birth date) significantly affected their reproductive productivity, rendering higher fertility rates and heavier calves when the yearling hind’s weight was higher (earlier birth). In addition, our results also showed that, after the melatonin treatment, a low yearling hind’s weight (‘late’ hinds) ceases to have a negative effect on their reproductive productivity, increasing fertility (46%) and the calves’ weight (7.3 kg) compared to the non-treated group. Similarly, Asher et al. [17] also demonstrated the effect of exogenous melatonin in lowering the critical threshold of body mass to puberty attainment. These authors pointed out that melatonin treatment would produce an effect on the body conditions of the yearling hind (at the muscle and fat level) at mating time. They suggested that melatonin can influence various components of the seasonal physiology of the red deer [31], modifying the relationship between body mass and composition [17]. Thus, yearling hinds with similar body mass could have a different ratio of muscle to fat.

On the other hand, the melatonin-mediated increase of the calves’ weight would shorten their fattening-up periods, allowing the farmer to meet the demand of this market by slaughtering them in the early spring (9–11 months old). Therefore, it would be of great interest to cause births to occur earlier, and to maximize the growth rates in the first months of life, before winter starts. In this context, melatonin implants should be considered to increase the calves’ weight before winter, since keeping the animals throughout this season (when the cost of feeding is higher) involves a significant economic cost [30].

Finally, the administration of exogenous melatonin markedly improved the reproductive productivity of the yearling hinds, increasing the number of those calved. Melatonin could facilitate such an improvement by furthering their first ovulation, as has been previously reported in deer [28,31], increasing conception at their first estrus. Moreover, it is possible that those hinds displaying their first estrus earlier within the rutting period potently boosted the stag mating activity, which in turn resulted in the enhancement of the fertility rates and advancement in calving observed in our study. In this sense, it has been reported that social interactions can prompt reproductive behavior in deer (i.e., ovulation induction is possible by male effect [32,33] as well as by female-to-female stimulation [25]). Our results showed that, though the introduction of the stags to the non-treated group of hinds did not allow us to achieve the fertility rates reached with the melatonin treatment, mixing both groups of hinds enhanced the fertility rates of the non-treated yearling hinds up to the values of the melatonin-treated hinds (≈78%). Presumably, there was a more effective induction of ovarian activity in the yearling hinds exposed to the melatonin treated hinds than that prompted by the introduction of the stags in the non-treated group. In line with this, Wilson [34] reported that the male effect applied in red deer farms only achieved half of the advances in the reproductive season of hinds compared to other treatments (exogenous melatonin, use of progesterone and gonadotropins). Such female-to-female stimulation of the hypothalamus–pituitary axis might be facilitated by visual, auditory or olfactory cues, as in other ruminant species, such as cows, the exposure of heifers to bovine cervical mucus collected at estrous caused an ovarian activity induction of the females [35]. To the best of our knowledge, this is the first time that the social facilitation effect of melatonin-treated yearling hinds on non-treated ones has been reported (stags did not receive melatonin implants). Different researchers [28,32] observed a similar effect on non-treated hinds (adults and yearling); however, in both studies, it was not possible to distinguish between the inductive effects of melatonin-treated stags or hinds. Our finding is relevant for the Iberian red deer farming industry, as melatonin treatments can simplify the increase of the reproductive productivity of the yearling hinds and reduce farm inputs.

## 5. Conclusions

In summary, melatonin implants, administered three-fold (two implants each time) every 30 days before the breeding season in yearling Iberian red deer hinds was positively associated to reproductive productivity. In addition, melatonin implants represent a very useful management tool to reduce the constraint effect of yearling hinds’ low weight on their future reproductive performance. Finally, we demonstrated the positive effect on fertility rates that melatonin-treated yearling hinds exert on non-treated hinds, which allows us to reduce the number of melatonin implants used, and thus reduce the cost when this protocol is applied in deer farms.

## Figures and Tables

**Table 1:** Distribution of the sample size across the years of study.

Year	Melatonin	Control
1	53	75
2	138	28
3	108	86
4	112	32
5	87	19
6	82	42
7	64	10
8	65	59
9	109	20
10	43	41
11	46	31
Total	907	443

**Table 2:** Adjusted (AOR) and unadjusted (OR) odds ratios of fertility from Iberian yearling hind weight and treatment with melatonin implants.

Parameter	Yearling Hind Weight		Melatonin Treatment	
	OR (95% CI)	AOR (95% CI)	OR (95% CI)	AOR (95% CI)
Fertility	1.03 (1.00–1.05) *	1.02 (0.99–1.05)	1.54 (1.06–2.23) *	1.46 (1.01–2.13) *

* *p* < 0.05; OR: Odds Ratio; AOR: Odds Ratio adjusted for both factors (yearling hind weight and melatonin implant treatment).

## Data Availability

The data presented in this study are available on request from the corresponding author.
